# H_2_S-Generating Cytosolic L-Cysteine Desulfhydrase and Mitochondrial D-Cysteine Desulfhydrase from Sweet Pepper (*Capsicum annuum* L.) Are Regulated During Fruit Ripening and by Nitric Oxide

**DOI:** 10.1089/ars.2022.0222

**Published:** 2023-07-17

**Authors:** María A. Muñoz-Vargas, Javier López-Jaramillo, Salvador González-Gordo, Alberto Paradela, José M. Palma, Francisco J. Corpas

**Affiliations:** ^1^ Group of Antioxidants, Free Radicals and Nitric Oxide in Biotechnology, Food and Agriculture. Estación Experimental del Zaidín (Spanish National Research Council, CSIC), Granada, Spain.; ^2^ Instituto de Biotecnología, Department of Organic Chemistry, University of Granada, Granada, Spain.; ^3^ Proteomics Core Facility, Centro Nacional de Biotecnología, CSIC, Madrid, Spain.

**Keywords:** cysteine desulfhydrase, ripening, hydrogen sulfide, nitration, post-translational modification, pyridoxal 5′-phosphate

## Abstract

**Aims:**

Pepper fruit is a horticultural product worldwide consumed that has great nutritional and economic relevance. Besides the phenotypical changes that undergo pepper fruit during ripening, there are many associated modifications at transcriptomic, proteomic, biochemical, and metabolic levels. Nitric oxide (NO) and hydrogen sulfide (H_2_S) are recognized signal molecules that can exert regulatory functions in diverse plant processes. This study aims at analyzing the interrelationship between NO and H_2_S during fruit ripening.

**Results:**

Our data indicate that the H_2_S-generating cytosolic L-cysteine desulfhydrase (LCD) and the mitochondrial D-cysteine desulfhydrase (DCD) activities are downregulated during ripening but this effect was reverted after NO treatment of fruits.

**Innovation and Conclusion:**

Using as a model the non-climacteric pepper fruits at different ripening stages and under an NO-enriched atmosphere, the activity of the H_2_S-generating LCD and DCD was analyzed. LCD and DCD activities were downregulated during ripening, but this effect was reverted after NO treatment of fruits. The analysis of LCD activity by non-denaturing polyacrylamide gel electrophoresis (PAGE) allowed identifying three isozymes designated CaLCD I to CaLCD III, which were differentially modulated by NO and strictly dependent on pyridoxal 5′-phosphate (PLP). *In vitro* analyses of green fruit samples in the presence of different compounds including NO donors, peroxynitrite (ONOO^−^), and reducing agents such as reduced glutathione (GSH) and L-cysteine (L-Cys) triggered an almost 100% inhibition of CaLCD II and CaLCD III. This redox adaptation process of both enzymes could be cataloged as a hormesis phenomenon. The protein tyrosine (Tyr) nitration (an NO-promoted post-translational modification) of the recombinant LCD was corroborated by immunoblot and by mass spectrometry (MS) analyses. Among the 11 Tyr residues present in this enzyme, MS of the recombinant LCD enabled us to identify that Tyr82 and Tyr254 were nitrated by ONOO^−^, this occurring near the active center on the enzyme, where His237 and Lys260 together with the cofactor PLP are involved. These data support the relationship between NO and H_2_S during pepper fruit ripening, since LCD and DCD are regulated by NO during this physiological event, and this could also be extrapolated to other plant species.


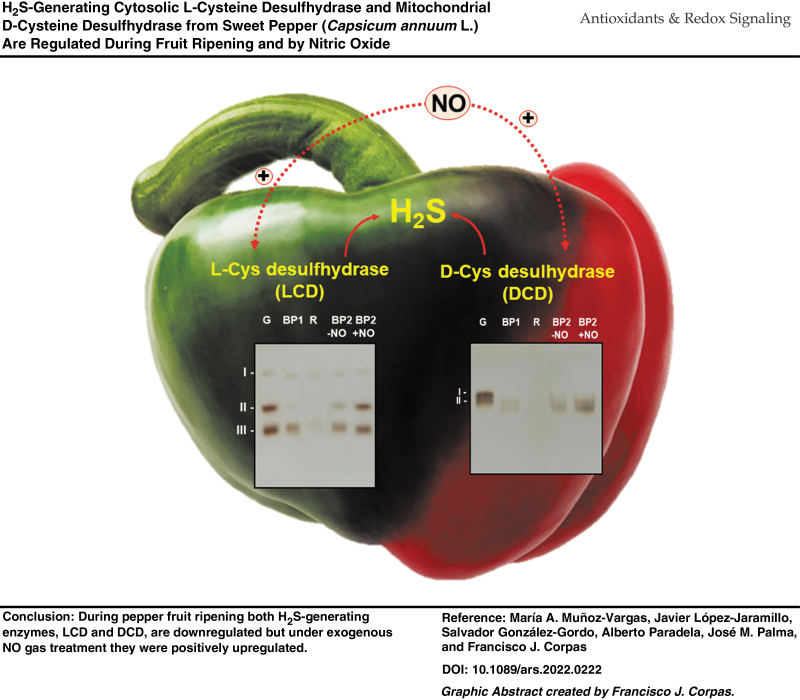


## Introduction

Hydrogen sulfide (H_2_S), which was previously considered a toxic molecule for living organisms, has now become a molecule of great interest due to its ability to modulate physiological and stress processes in both animal and plant systems (Abe and Kimura, 1996; Cirino et al., 2023; Corpas and Palma, 2020; Jurado-Flores et al., 2021). In the model plant *Arabidopsis thaliana*, H_2_S is generated through the cysteine (Cys) metabolism by several enzymes, including the cytosolic L-cysteine desulfhydrase (LCD) and L-cysteine desulfhydrase 1 (DES1); the chloroplastic sulfite reductase (SiR, EC 1.8.7.1); the mitochondrial bifunctional D-cysteine desulfhydrase/1-aminocyclopropane-1-carboxylate deaminase (DCDES1), D-cysteine desulfhydrase 2 (DCDES2) (Álvarez et al., 2010; Asada, 1967; Corpas et al., 2019; González-Gordo et al., 2020a), and β-cyanoalanine synthase (García et al., 2010).

The LCD is considered the main source of H_2_S and catalyzes the following reaction: L-cysteine + H_2_O → pyruvate + NH_4_^+^ + H_2_S + H^+^, requiring pyridoxal 5′-phosphate (PLP) as a cofactor (Kurmanbayeva et al., 2022; Muñoz-Vargas et al., 2022). LCD participates in diverse physiological processes such as root development (Mei et al., 2019), stomatal closure (Ma et al., 2021; Scuffi et al., 2014; Shen et al., 2020), leaf senescence (Hu et al., 2021), fruit ripening (Hu et al., 2020; Muñoz-Vargas et al., 2018), but also in the mechanism of response to diverse environmental stresses (Kaya, 2020; Kharbech et al., 2022; Sun et al., 2022; Wang et al., 2022; Wang et al., 2023; Zhou et al., 2021).

The relevance of H_2_S arises from its mediation in a post-translational modification (PTM) called persulfidation, which involves the thiol groups of amino acids, peptides, and proteins and that can either positively or negatively affect their function (Corpas et al., 2021a).

Nitric oxide (NO) is another molecule that was initially considered toxic since it was part of polluting episodes such as acid rain or the destruction of the ozone layer. However, currently, NO and derived molecules called reactive nitrogen species (RNS), such as peroxynitrite (ONOO^−^), nitrogen dioxide (NO_2_), and *S*-nitrosoglutathione (GSNO), can display regulatory functions in plants, mainly throughout PTMs such as *S*-nitrosation, tyrosine (Tyr) nitration, and metal nitrosylation (Corpas et al., 2020; Corpas et al., 2013; Gupta et al., 2020). Although the enzymatic source of NO in higher plants is still a subject of debate (Corpas et al., 2022a), it has been shown that NO and other RNS are involved in almost all the physiological processes of plants and the response mechanisms against abiotic and biotic stresses (Asgher et al., 2017; Bhat et al., 2021; Corpas et al., 2011; Kolbert et al., 2019).

Fruit ripening is a complex process that involves multiple changes at metabolic, proteomic, and transcriptomic levels that are regulated by different phytohormones (ethylene, ABA, *etc.*), as well as by different signal compounds including NO and, more recently, other molecules such as hydrogen sulfide (H_2_S) or melatonin (Aghdam et al., 2021b; Corpas and Palma, 2020). All these changes affect the organoleptic properties of fruits such as aroma, flavor, color, texture, and nutritional quality.

Pepper (*Capsicum annuum* L.) is a non-climacteric fruit that has great relevance at nutritional and economical levels. Thus, pepper fruit is an important source of vitamins C, A, and E, minerals, flavonoids, and carotenoids with recognized antioxidant properties (Guevara et al., 2021; Hernández-Pérez et al., 2020). Previous studies at biochemical, transcriptomic, proteomic, and subcellular levels indicate that the California-type sweet pepper fruit undergoes an active reactive oxygen species and RNS metabolism during ripening from immature green to ripe red stages (Chu-Puga et al., 2019; Corpas et al., 2018; González-Gordo et al., 2023; González-Gordo et al., 2022a; González-Gordo et al., 2022b; González-Gordo et al., 2022c; González-Gordo et al., 2020b; González-Gordo et al., 2019; Palma et al., 2018).

This work aims at investigating at the biochemical level two sources of H_2_S, the cytosolic LCD and the mitochondrial D-cysteine desulfhydrase (DCD), during the ripening of sweet pepper fruits, and evaluating their interaction with NO. Taken together, the data obtained indicate that these two enzymes are modulated by NO during maturation, where LCD is inhibited by tyrosine nitration events.

## Results

Based on the information available in the Arabidopsis database (TAIR) of the genes that code for enzymes involved in the H_2_S biosynthesis in the different subcellular compartments, the identification of their corresponding orthologous in the pepper fruit transcriptome (Sequence Read Archive, SRA) was carried out, and the data are available at https://www.ncbi.nlm.nih.gov/sra/PRJNA668052. Likewise, the *C. annuum* proteome was deposited at https://mobidb.org/P86005, reference proteome UP000189700. Table 1 shows the identified proteins that can generate H_2_S from the two cysteines (Cys) isomers, either L-cysteine (L-Cys) or D-Cys. Thus, we focus on the cytosolic LCD and the mitochondrial DCD that are considered the main sources of H_2_S in higher plants (Mei et al., 2019; Muñoz-Vargas et al., 2022; Scuffi et al., 2014; Yamasaki et al., 2019).

**Table 1.** Identified Enzymes Involved in the Generation of Hydrogen Sulfide in Pepper Fruit

Protein name	Acronym	Capsicum annuum			Arabidopsis thaliana			% ID	Subcell. location
		UniProtKB ID	No. AA	MW (kDa)	TAIR ID	No. AA	MW (kDa)		
L-cysteine desulfhydrase	LCD	A0A2G3AJZ6	453	50.3	At3g62130	454	50.7	62.1	Cyt
Cysteine synthase	DES1	A0A1U8G3Y0	325	34.4	At5g28030	323	34.3	69.7	Cyt
L-cysteine desulfhydrase	CDES	A0A1U8GPK5	426	47.4	At5g26600	475	52.9	63.8	Pla
D-cysteine desulfhydrase 1	DCDES1	A0A1U8FXQ2	425	46.7	At1g48420	401	43.9	72.2	Mit
D-cysteine desulfhydrase 2	DCDES2	A0A2G3AI42	448	49.4	At3g26115	427	47.4	55.9	Mit

% ID, percentage of identity in comparison to the corresponding Arabidopsis protein; AA, amino acids; Cyt, cytosol; LCD, L-cysteine desulfhydrase; Mit, mitochondrion; MW, molecular weight; Pla, plastid.

### LCD and DCD activities during pepper fruit ripening and under exogenous NO gas treatment

**FIG. 1.**
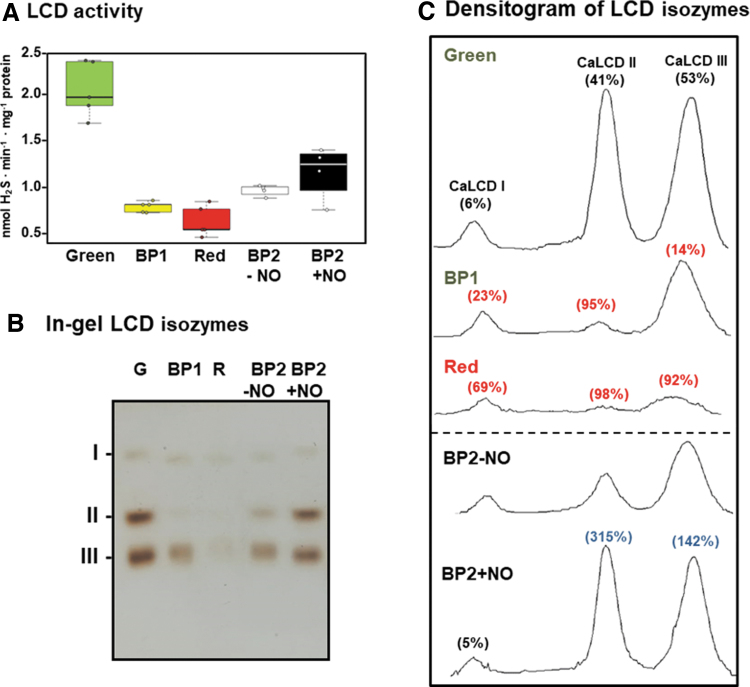
**LCD activity of sweet pepper fruits at different ripening stages: immature green (G), BP1, breaking point 2 with and without NO treatment (BP2 + NO and BP2 − NO, respectively), and red ripe (R) (see Supplementary Fig. S1 for the experimental design). (A)** Box plot showing the spectrophotometric assay of total LCD activity. Data are means ± SEM of three independent samples. **(B)** In-gel isozyme profile of LCD activity. Protein samples (45.5 μg per lane) were separated by non-denaturing PAGE (8% acrylamide), and activity was detected by the lead acetate staining method using L-Cys as substrate and PLP as cofactor. LCD isozymes were labeled I–III. **(C)** Densitometric analysis of LCD isozymes and their relative quantification (%) made by the ImageJ 1.45 program according to the isozyme profiles obtained in **(B)**. The numbers in parentheses indicate the percentage of either inhibition (*red*) or activation (*blue*) in each condition. The picture and densitometric analyses of the gel are representative of at least three biological replicates. BP, breaking point; L-Cys, L-cysteine; LCD, L-cysteine desulfhydrase; NO, nitric oxide; PAGE, polyacrylamide gel electrophoresis; PLP, pyridoxal 5′-phosphate; SEM, standard error of the mean.

Figure 1A shows the biochemical analysis of the cytosolic H_2_S-generating LCD in pepper fruits at different ripening stages: immature green (G), breaking point (BP1), BP2 with and without NO (BP2 + NO and BP2 − NO, respectively), and ripe red (R), as previously characterized (Supplementary Fig. S1) (González-Gordo et al., 2022b). Thus, the LCD activity showed a threefold decrease in red fruits compared with green ones, whereas the activity profile displayed intermediate values both with and without NO treatment. On the other hand, NO caused a 34% increase in LCD activity in comparison to untreated plants (BP2 + NO *vs.* BP2 − NO).

As part of the biochemical characterization of the LCD enzyme system in pepper fruits, the presence of potential isozymes was also analyzed by non-denaturing PAGE during fruit ripening and under NO treatment. Figure 1B displays the identification of three LCD isozymes designated CaLCD I to CaLCD III, according to their increasing electrophoretic mobility in the gel, and it was observed that they were differentially modulated during ripening and by NO.

Figure 1C depicts the total relative activity quantification considering all the H_2_S-generating LCD isozymes present at each ripening stage. In green pepper, CaLCD I isozyme accounted for 6% of the total activity, whereas CaLCD II and CaLCD III represented 41% and 53%, respectively (Fig. 1C). After ripening, the band intensities of CaLCD II and CaLCD III were significantly diminished about 98% and 92%, respectively.

Under an NO environment (BP2 + NO), a positive effect was observed on these two isozymes, with 315% increases for CaLCD II and 142% for CaLCD III. These results were in good agreement with the data observed in the spectrophotometric assays of total LCD activity (Fig. 1A).

**FIG. 2.**
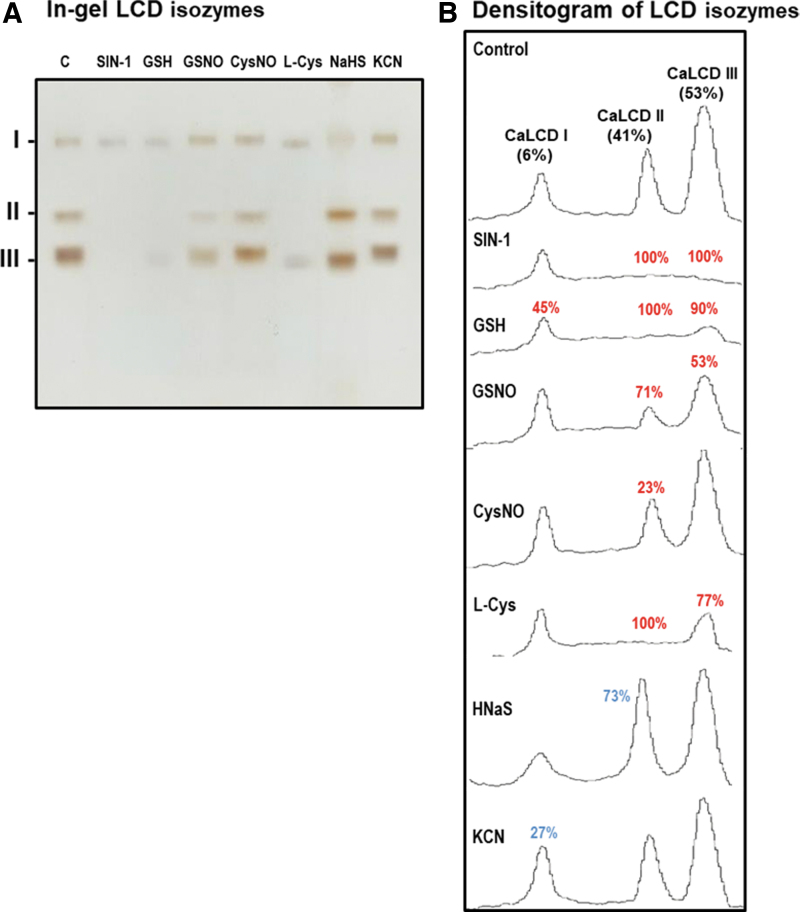
**Effect of nitration, *S*-nitrosation, and reducing agents on the *green* pepper fruit LCD isozymes analyzed in non-denaturing gels. (A)** In-gel isozyme profile of LCD activity in 8% acrylamide gels. **(B)** Densitometric analysis of LCD isozymes and their relative quantification (%) was made by the ImageJ 1.45 program. SIN-1, is a ONOO^−^ donor and a nitrating compound. GSNO and CysNO are NO donors and nitrosating agents. L-Cys, cysteine; HNaS, sodium hydrosulfide as H_2_S donor. All treatments were done by pre-incubating the green pepper samples (80 μg protein per lane) with these compounds (5 m*M*) at 25°C for 1 h, except with SIN-1, which was pre-incubated at 37°C for 1 h. The number assigned to each peak indicates the percentage of either isozyme activity inhibition (*red*) or activation (*blue*) in relation to the control samples (green fruit crude extracts) after the quantification made with the help of the ImageJ 1.45 program. The picture and densitometric analyses of the gel are representative of at least three biological replicates. CysNO, *S*-nitrosocysteine; GSH, reduced glutathione; GSNO, *S*-nitrosoglutathione; H_2_S, hydrogen sulfide; KCN, potassium cyanide; ONOO^−^, peroxynitrite; SIN-1, 3-morpholinosydnonimine.

To gain deeper insights into how each specific LCD isozyme might be influenced by the NO treatment, *in vitro* analyses of different potential modulators on the activity of the three LCD isozymes identified in green pepper fruits were carried out by non-denaturing PAGE (Fig. 2A). The relative quantification of each LCD isozyme showed that the CaLCD III, which is the most prominent isozyme, as shown earlier, was inhibited by 100% by the nitrating agent 3-morpholinosydnonimine (SIN-1), an ONOO^−^ donor, 90% by reduced glutathione (GSH), 77% by L-Cys, 53% by the NO donor GSNO (a nitrosating compound), and 11% by potassium cyanide (KCN); however, sodium hydrosulfide (NaHS) did not affect this isozyme (Fig. 2B).

CaLCD II isozyme was inhibited by 100% with SIN-1, GSH, and L-Cys, about 71% by GSNO, and by 23% by L-Cys NO. On the contrary, the activity of CaLCD II was increased by 73% by NaHS and 14% by KCN. CaLCD I was inhibited by 45% after incubation with GSH, 43% by SIN-1, 27% with HCN, 15% with GSNO, 8% with L-Cys, and 7% with NaHS. Oppositely, this isozyme activity was increased by 27% in the presence of KCN.

**FIG. 3.**
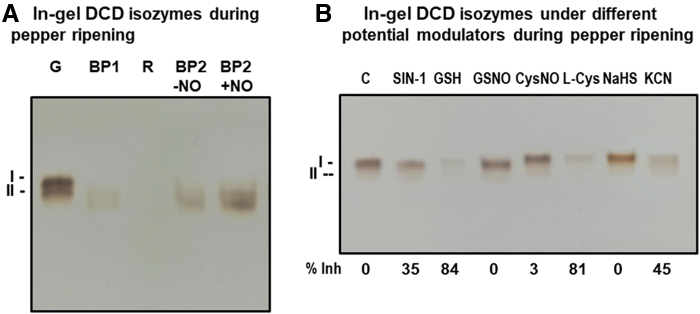
**In-gel DCD activity of sweet pepper fruits.** Non-denaturing PAGE on 6% acrylamide gels was carried out, and activity was detected by the lead acetate staining method using D-Cys as substrate and PLP as cofactor. **(A)** DCD activity in fruits at different ripening stages: immature green (G), BP1, BP2 with and without NO treatment (BP2 + NO and BP2 − NO, respectively), and red ripe (R). **(B)** Effect of nitration (SIN-1), *S*-nitrosation (GSNO, CysNO), and reducing agents (GSH, L-Cys) on green pepper fruit DCD isozymes analyzed in non-denaturing acrylamide gels. The *numbers below* indicate the percentage of inhibition (% Inh) in relation to the control samples (C), and this was quantified by the ImageJ 1.45 program. The pictures are representative of at least three biological replicates. DCD, D-cysteine desulfhydrase.

In the case of the mitochondrial DCD, an analysis through non-denaturing PAGE of potential isozymes was also accomplished (Fig. 3A). Two isozymes with close mobilities were distinguished and designated DCD I and DCD II, with DCD I being the slower and the most prominent isozyme. Both DCD isozymes decreased drastically during ripening and were nearly undetectable in red ripe fruits. When the fruits at BP2 were exposed to NO gas, an overall increase in the two-isozyme activity was observed.

Likewise, to get deeper insights into the post-translational regulation of these DCDs, *in vitro* assays were performed using green pepper samples pretreated with the same modulators used for the LCD assays (Fig. 3B). ONOO^−^ caused a 35% inhibition: The NO donors GSNO and *S*-nitrosocysteine (CysNO) had almost no effect on the activity, whereas the reductants GSH and L-Cys provoked an inhibition by 84% and 81%, respectively.

On the other hand, the H_2_S donor did not affect the activity whereas the KCN caused inhibition of 45%. Therefore, the data suggest that DCD is susceptible to undergo nitration and *S*-nitrosation, and, the later, it seems to protect the activity from the inhibition triggered by GSH and L-Cys. As an internal control, when the D-Cys substrate was not added to the reaction mixture, no activity band was detected.

**FIG. 4.**
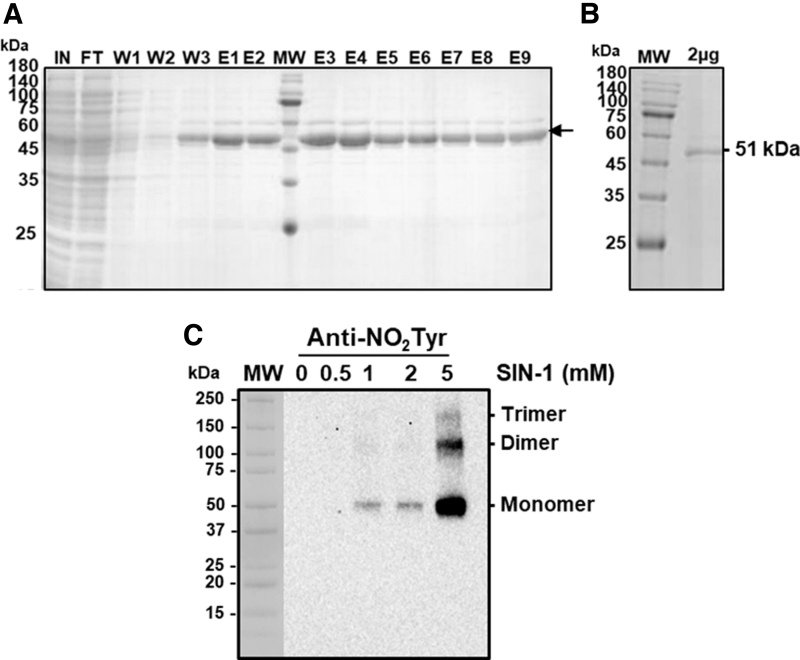
**Purification of recombinant LCD and effect of SIN-1 (ONOO^−^ donor**)**. (A)** SDS–PAGE on 4%–20% acrylamide gels analysis of the recombinant LCD purification from sweet pepper fruits stained with Coomassie blue. Fractions were obtained after LYTRAP affinity column chromatography. E1–E9, eluted fractions; MW, MW markers; W1–W3, washing fractions. **(B)** Recombinant LCD (2 μg per lane) after buffer exchange and used for mass spectrometric analyses. **(C)** Representative immunoblot showing the degree of tyrosine nitration of recombinant LCD treated with different concentrations of SIN-1 and detected with an antibody against 3-nitrotyrosine (dilution 1:2000). A precast 4–20 SDS-PAGE was used and 0.5 μg of LCD protein was used per line. The secondary antibody was used at a concentration of 1:10,000. FT, flow through; IN, input; MW, molecular weight; SDS, sodium dodecyl sulfate.

Considering that SIN-1 caused the most relevant inhibitory effect on the most abundant isozymes (LCD II and III) and, as a mean of increasing the knowledge on the pepper LCD regulation mechanism, a recombinant LCD protein was obtained by sequencing the pepper clone and overexpressing it in *Escherichia coli*. Figure 4A and B show the sodium dodecyl sulphate SDS-PAGE analysis of the different fractions obtained after LYTRAP affinity column chromatography (Biomedal, Ltd., Sevilla, Spain).

The recombinant cytosolic LCD protein showed a size of 51 kDa, which is in line with the expected theoretical molecular mass of this protein. Fractions E3–E9 showed an adequate purity grade for this protein (Fig. 4A). Unfortunately, the obtained recombinant protein was not active, so its specific activity could not be tested against any potential modulator even after attempting its reconstitution with 25 m*M* HEPES buffer at pH 7.5 containing 50 m*M* NaCl, 50 μ*M* PLP, and 10 m*M* β-mercaptoethanol with an overnight incubation at 4°C.

Alternatively, immunoblot assays with an antibody against nitrotyrosine (NO_2_-Tyr) were carried out. Thus, the recombinant LCD protein was incubated with increasing concentrations of SIN-1 (0, 0.5, 1, 2, and 5 m*M*) previous to SDS-PAGE and then analyzed by immunoblot assay with the antibody against NO_2_-Tyr. Figure 4C shows an immunoreactive band of 51 kDa, detected after pre-incubation with 1–5 m*M* SIN-1. It is remarkable that with the highest concentration (5 m*M*), two additional immunoreactive bands were observed, which seemed to correspond with the formation of dimer and trimer forms, with the monomeric one being the most immuno-reactive band.

**FIG. 5.**
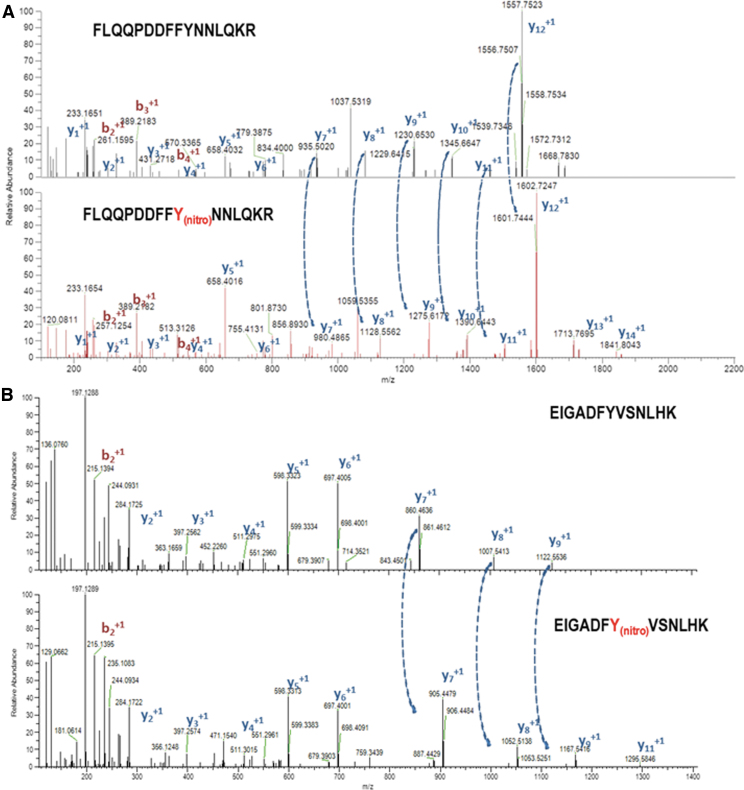
**Identification of the tyrosine residues of the pepper LCD susceptible to be nitrated by mass spectrometric.** Mass spectrometry fragmentation spectra corresponding to the unlabeled (*upper panel*) and labeled (Y10-nitro,*lower panel*) version of peptides **(A)** FLQQPDDFFYNNLQK and **(B)** EIGADFYVSNLHK polypeptides contain the nitrated Tyr82 and Tyr254, respectively, of the sweet pepper LCD. The fragments corresponding to the main fragmentation series “b,” if the charge is retained on the N-terminus (*red*) and by “y,” if the charge is maintained on the C-terminus (*blue*). The *subscript* in both series (b_*n*_, y_*n*_) indicates the number of amino acid residues in the considered fragment from either N-terminus or C-terminus. The superscript refers to the charge (+1) of the backbone fragmentation. *Dotted arrows* connecting both graphs in **(A, B)** indicate the increase of +44,985 Da corresponding to the modification Y10 → Y10_nitro_ and Y7 → Y7_nitro_, respectively. Tyr, tyrosine.

**FIG. 6.**
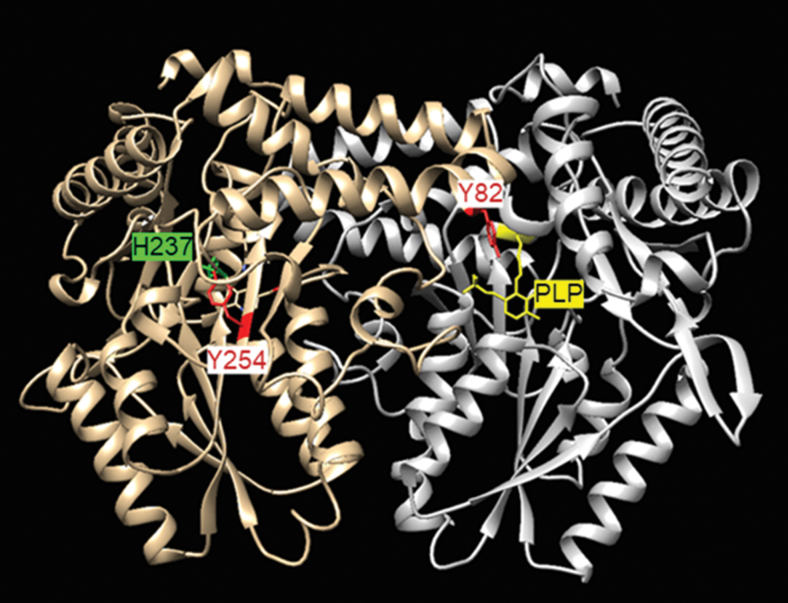
**Localization of nitrated tyrosine in the three-dimensional model of the pepper LCD (CaLCD; UniProtKB entry A0A1U8DYD9) showing the position of the nitrated tyrosines (Tyr82 and Tyr254) and their potential interactions with His237 and PLP in yellow**.

To identify which of the 11 Tyr present in the pepper LCD is(are) target(s) of this PTM, non-nitrated and ONOO^−^-treated recombinant LCD were subjected to trypsin digestion followed by matrix-assisted laser desorption/ionization-time-of-flight (MALDI-TOF/TOF) mass spectrometry (MS) examination (Fig. 5). Among the identified peptides, two of them containing nitrated tyrosine within the LCD amino acid sequences FLQQPDDFFYNNLQKR and EIGADFYVSNLHK with masses of 2118.27 Da (2073.27 + 45 Da) and 1537.63 Da (1492.63 + 45 Da) were compatible with the acquisition of a nitro (-NO_2_) group (-NO_2_ 45 Da) on Tyr82 and Tyr254, respectively. Figure 6 shows the localization of these nitrated tyrosines in the three-dimensional model of the pepper LCD, that are close to His237 and PLP.

### Modeling of the pepper LCD

The modeling of the structure of LCD from *C. annuum* L. (UniProtKB entry A0A1U8DYD9) was approached by homology modeling at five different servers. Whereas the Swiss Model (Arnold et al., 2006) and M4T (Fernandez-Fuentes et al., 2007) computed a single model, I-Tasser (Zhang, 2008), Phyre2 (Kelley and Sternberg, 2009), and Raptor X (Källberg et al., 2012) output several models, and only those with the better scores were considered.

The fact that scoring functions are server-dependent makes unfeasible the direct scoring of models generated by different servers. For this reason, the quality of the models was assessed in terms of the distribution of atom-atom contacts (Errat method) (Colovos and Yeates, 1993), three-dimensional profiles (Verify3D) (Eisenberg et al., 1997), stereochemical quality (Procheck) (Laskowski et al., 1993), and a composite scoring function (Qmean4 and Qmean-Z score) (Benkert et al., 2011; Benkert et al., 2008).

As depicted in Supplementary Table S1, the model computed by the Swiss Model and model #5 from RaptorX showed the best parameters of quality and shared similar tertiary structure, with a root mean square deviation (RMSD) of 1.189 Å over 219 Cα pairs, and some discrepancies in loop regions (Supplementary Fig. S2).

The model generated by the Swiss Model was used as a template for the PDB entry 5uts, which shares 27% identity with LCD from *C. annuum* L. and corresponds to a PLP-dependent C-S lyase Egt2, involving sulfoxide C-S bond cleavage in the ergothioneine biosynthesis, a trimethylated and sulfurized histidine derivative that exhibits antioxidant properties (Irani et al., 2018; Sheridan et al., 2016). This model was considered superior because it consists of the dimeric organization described in other desulfurases and our studies were focused on it.

However, it did not include the cofactor, which is expected to form an internal aldimine (*i.e*., Schiff base) between the carbonyl group of PLP and lysine in the active site. The structural superposition of the model and template yields an RMSD of 0.260 Å over 345 Cα pairs and allowed the identification of Lys260 as the residue that forms the aldimine (Supplementary Fig. S3A). The model was manually modified to include PLP and generate the bond.

The analysis at PLIP sever (Adasme et al., 2021) revealed that the cofactor, besides forming a Schiff base with Lys260, establishes hydrophobic interactions with Ala236, π-stacking with Phe147, hydrogen bonds with Asn113, Ala114, Thr115, His237, Asn257, and Phe304T, and salt bridges with His259 (Supplementary Fig. S4). In 5uts, residues Phe48 and His276 interact with the substrate and they match with Phe55 and His276 in the model of LCD, but Arg75 and Phe304, which have been described as involved in the stabilization of the ergothioneine sulfenic acid intermediate, are not coincident.

Given the facts that some servers used as template cysteine desulfurases (Supplementary Table S1), that Cys58, Cys145, Cys211, and Cys264 are found within 15 Å from the cofactor (Supplementary Fig. S5A), and that their positions are coincident with those predicted by model 5 computed at Raptor X (Supplementary Fig. S5B), their potential role as the nucleophilic Cys was evaluated. The identification of the catalytic Cys was approached by structural alignment with the type II structures from *A. thaliana* (PDB entry 4q75) (Roret et al., 2014), *Bacillus subtilis* (PDB entry 5zs9) (Nakamura et al., 2020), and *E. coli* (PDB entries 1c0n and 1kmj) (Fujii et al., 2020; Lima, 2002).

The match between the LCD model and the crystallographic structures yields RMSD values ranging from 1.188 Å (160 Cα pairs) for the enzyme from *B. subtilis* to 1.313 (171 Cα pairs) for the enzyme from *A. thaliana*. However, none of the four cysteines from LCD matched the coincident position of the nucleophilic cysteine in the four crystallographic structures, with Cys145 and Cys211 being the closest to the ideal position (Supplementary Fig. S5C). In fact, the position of the putative reactive loop of LCD is far away from that of the *A. thaliana* enzyme and, more importantly, it does not bear any Cys residue (Supplementary Fig. S5D).

In addition, the four cysteines close to the cofactor were analyzed in terms of evolutionary conservation (rho value) (Mihalek et al., 2004) in green plants (*i.e*., clade Viridiplantae), accessibility (Kabsch and Sander, 1983), and predicted pKa (Li et al., 2005). As depicted in Supplementary Table S2, residue Lys260, as expected from its catalytic role, shows a low rho value (*i.e*., high evolutionary preservation) and a much lower predicted pKa than the typical value for the ɛ-amine group (*i.e*., pKa 10), in full agreement with its high nucleophilicity to yield the formation of the internal aldimine with the carbonyl group of the PLP, but none of the four cysteines near the cofactor is especially outstanding. The predicted pKa values are higher than that of a model cysteine sulfhydryl group (*i.e*., pKa 9), with Cys211 being the least preserved, but contributing to the anomalous pKa of Lys260, and Cys145 the most accessible.

Alternatively, to gain additional insight into the catalytic site of the pepper LCD, the model was docked with the substrate (L-Cys) and the product (pyruvate). Blind docking analysis found positive hints for both L-Cys and pyruvate, with the poses located in two preferential regions, one at Cys447 and another at Lys260 (Supplementary Fig. S6). Whereas Cys447 is far from the active site and its participation in the catalytic process can be discarded, the poses at the active site are significant, especially when considering that they are not biased by any restriction of the docking area, since the target was the entire protein molecule.

**FIG. 7.**
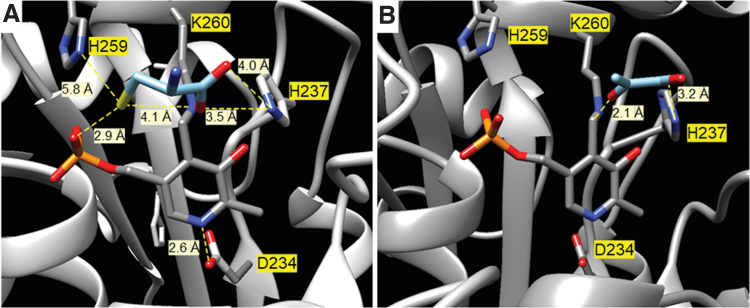
**Analysis of the positions at the active site of the pepper LCD.** Interactions of L-Cys **(A)** and pyruvate **(B)** at the active site of pepper fruit LCD as predicted by blind docking. These interactions were taken from the ZINC database and carried out at Swiss-Dock server in a precise mode, allowing flexibility for the side chains within 5 Å of any atom of the ligand in its reference binding mode, without defining the region of interest (blind docking).

A closer analysis of the positions at the active site revealed that L-Cys is at the hydrogen bond distance of the phosphate group of PLP and the imidazole ring of His237 and at 5.8 Å from His259 (Fig. 7A). Moreover, both the orientation of the keto group of the pyruvate and its distance from the carbonyl group is correct considering that it is the result of the hydrolysis of the enamine after the L-Cys desulfuration (Fig. 7B).

The predictions of the features of these residues (Supplementary Table S1) support their importance. The low rho values are indicative of high evolutionary preservation in the clade Viridiplantae, and the anomalous low values of their predicted pKa are expected for residues with a catalytic role (Harris and Turner, 2002).

## Discussion

NO and H_2_S are two molecules that have signaling functions in all aspects of plant physiology (Mishra et al., 2021) and can exert this role through reversible oxiPTMs (*S*-nitrosation and persulfidation), as well as by some irreversible processes such as protein nitration (Campolo et al., 2020; Corpas et al., 2022c; Corpas et al., 2021b) or *S*-sulfenylation (Beedle et al., 2016). Pepper fruits are in the category of non-climacteric fruits and are one of the most consumed horticultural products with great economic relevance.

Considering that the available information on how H_2_S is generated endogenously in fruit is very limited, this study aims at identifying and characterizing two of the enzymatic activities responsible for its generation, cytosolic LCD and mitochondrial DCD, and how they could be modulated during the ripening process as well as by the exogenous effect of NO gas (5 ppm/h).

### Endogenous enzymatic sources of H_2_S are downregulated during ripening but upregulated under an NO-enriched atmosphere

The enzymatic generation of H_2_S from L-Cys in leaves from different plant species was demonstrated in the early 1980s (Sekiya et al., 1982), and some years later its generation from D-Cys was also observed (Rennenberg et al., 1987). However, data on these enzymes in fruits and their functional implication are very scarce since most of the information comes from the model plant *A. thaliana* and some few crops but mainly focused on the mechanism of response against stressful conditions.

Thus, the upregulation of the LCD activity and gene expression is part of the mechanism of response against different environmental conditions such as manganese stress (Hou et al., 2022), hypoxia (Xuan et al., 2022), and chromium stress in maize (Kharbech et al., 2022) or cadmium stress in *Vigna radiata* (Khan et al., 2020). In rice subjected to drought stress, an inhibition of nitrate reductase (NIA2) activity was found, but the overexpression of LCD1 intensified the persulfidation of NIA2 and its activity (Zhou et al., 2021).

Very recently. it has been shown that the protein arginine methyltransferase 5 (AtPRMT5) can increase LCD activity through methylation modifications during Arabidopsis cadmium stress responses since the generated H_2_S reduced the negative impact of this metal (Cao et al., 2022).

However, in these previous studies, the isoenzymatic profile of LCD and DCD was not addressed. Recently, using non-denaturing PAGE, we analyzed different plant species such as ginger, leek, broccoli, and different *Allium* species and this allowed us to identify the presence of up to nine different LCD isozymes with leek and broccoli being the species with the highest number of LCD isozymes (Muñoz-Vargas et al., 2022).

Using this methodological approach, we have identified three LCD isozymes and two DCDs that were differentially modulated during the fruit ripening of sweet pepper. While LCD II and LCD III decreased drastically during ripening, LCD I remained mostly stable. Interestingly, NO gas exertes a positive modulating effect on LCD II and III, suggesting that these isozymes are susceptible to undergoing NO-derived PTMs such as *S*-nitrosation and/or nitration. Similar behavior was observed for the DCD isozymes.

These data manifest the functional cross-regulation on the metabolism of both molecules, NO and H_2_S, during pepper fruit ripening, and the decline of LCD and DCD isozymes during ripening and their upregulation by NO treatment support that NO seems to function as an upstream signal of the H_2_S metabolism. To our knowledge, this is the first report concerning the modulation of LCD and DCD isozymes during ripening and under an NO gas atmosphere.

The presence of D-stereoisomer of specific amino acids starts to attract the attention of researchers due to their physiological functions. In the case of D-Cys as an H_2_S source, it has been shown in mouse cells that D-Cys is present in the central nervous system where it modulates the neural progenitor cell homeostasis in the developing brain (Semenza et al., 2021).

Thus, D-Cys seems to regulate mammalian neurodevelopment by inhibiting the protein kinase B (PKB), also known as Akt, and appears to be involved in schizophrenia and Alzheimer's disease (Roychaudhuri and Snyder, 2022). An interesting case was observed in the water fern *Azolla pinnata*, where D-Cys induced root abscission in a wide variety of environmental stimuli (Yamasaki et al., 2019), suggesting that it could be a mechanism that allows freeing itself from the roots allowing some of the ferns to be transported to areas where growing conditions are more optimal.

Regarding the case of DCD activity in higher plants, it should be remarked that the information is even scarcer than that on the LCD. Although the existence of PLP-dependent DCD activity was demonstrated in different plant species some time ago (Rennenberg et al., 1987; Schmidt, 1987), the first isolation and characterization of this mitochondrial enzyme was achieved in Arabidopsis (Riemenschneider et al., 2005). It was demonstrated that the *DCD* gene expression increased during development.

However, during senescence, whereas the protein expression remained unaffected, the DCD activity was the highest. It is remarkable that in our case, during fruit ripening, which could be considered a senescent process, we find a dissimilar behavior, since the activity of both DCD isozymes decreased significantly. In addition, in the analysis of Arabidopsis DCD activity in plants grown under short-day conditions (12-h light/12-h dark), a lower activity during the dark phase was found, thus suggesting the implication of PTMs (Riemenschneider et al., 2005).

More recently, additional studies have started to correlate DCD activity with stress conditions. For example, in Arabidopsis under cadmium stress, the DCD activity seems to promote tolerance to this metal (Zhang et al., 2020). In a study using broccoli during postharvest cold storage that usually triggers floret yellowing, it was shown that the application of phytosulfokine-α (a disulfidated peptide growth factor) delayed the senescence due to an increase of the H_2_S content as a consequence of the simultaneous enhancement of LCD and DCD activities (Aghdam et al., 2021a).

### *In vitro* pharmacological analysis of LCD and DCD activity shows that NO exerts dual effects

To gain a deeper knowledge of the potential regulation of LCD and DCD by NO, we performed *in vitro* analyses applying the pre-treatment of green pepper samples with different compounds with the capacity to release either ONOO^−^ (SIN-1) or NO (GSNO and NO-Cys), as well as with H_2_S and cyanide, which is a recognized inhibitor of different enzymes such as the antioxidant enzymes CuZnSOD and the mitochondrial electron transport chain at the level of the cytochrome *c* oxidase (complex IV).

Thus, green pepper samples pre-treated with ONOO^−^ underwent a drastic reduction of the LCD isozymes II and III, whereas the DCD was diminished more slightly. The treatment with the NO donors apparently did not affect the activity of the different LCD and DCD isozymes. However, when compared with the used internal controls, both GSH and L-Cys triggered a significant reduction in their activity. Thus, it could be deduced that these two reducing agents exert a negative effect on these activities but, in the presence of NO, the respective activity remains, suggesting that the enzymes undergo a process of S-nitrosation to protect against the inhibitory effect observed by GSH (*S*-glutathionylation) and L-Cys.

Remarkably, neither the H_2_S donor NaHS nor the cyanide provoked significant effects on these activities. Therefore, the data indicate that LCD activity is well regulated by both its own substrate (L-Cys) and NO (nitration and S-nitrosation). Interestingly, the inhibition of the LDC by high L-Cys concentrations could have a physiological significance considering that high concentrations of H_2_S are toxic for the cells, being a mechanism of autoregulation to avoid a cellular overproduction of this compound.

This observed redox adaptation processes of the LCD and DCD could be cataloged as a hormesis phenomenon that allows to improve its functionality in response to redox changes in the surrounding cellular environment of these enzymes (Calabrese and Mattson, 2017; Calabrese et al., 2010).

### Nitration of Tyr82 and Tyr254 of LCD seem to interfere with the binding of pyridoxal phosphate and triggers the inhibition of the catalysis

Nitration is a PTM mediated by different RNS, which involves the addition of a nitro group (-NO_2_) to some susceptible amino acids such as Tyr or tryptophan (Trp). Usually, this PTM triggers a loss of function (Corpas et al., 2021b; Ferrer-Sueta et al., 2018). In our case, the pretreatment of pepper samples with the ONOO^−^ donor SIN-1 provoked a diminishment of the enzyme activity of LCD isozymes II and III; however, in the case of DCD, this effect was less pronounced, suggesting that the affected Tyr should be less critical for the activity.

To our knowledge, this is the first report showing this effect in the cytosolic LCD, suggesting that NO can function upstream of H_2_S. In Arabidopsis, it has been shown that LCD can undergo persulfidation as a mechanism of regulation of stomata movement (Shen et al., 2020). However, to the best of our knowledge, it has not been previously reported that nitration affects either LCD or DCD activities.

To better understand how this PTM operates with the pepper fruit desulfhydrases, we obtained the recombinant pepper LCD protein and it was exposed to increasing concentrations of SIN-1 (0.5–5 m*M*). Thus, with 1 m*M* SIN-1, an immunoreactive band corresponding to the monomer of the LCD was detected. However, with 5 m*M* SIN-1, two additional bands of higher sizes were observed, and they corresponded to the dimer and trimer forms according to their molecular masses, around 100 and 150 kDa, respectively.

This behavior is not unusual since nitration, as well as oxidation, can provoke protein misfolding and aggregation in animal samples (Giasson et al., 2000; Kummer et al., 2011; Matalon et al., 2009; Nakamura et al., 2021). This will support the loss of activity observed in our samples, although to our knowledge, there is no information on this issue, but it could explain the inhibition of LCD activity under nitro-oxidative stress conditions, such as it has been previously described in pepper fruits during their ripening (Chaki et al., 2015; González-Gordo et al., 2020b).

The data obtained by mass spectrometric analysis allowed us to identify that, among the 11 Tyr present in the LCD, Tyr82 and Tyr254 were nitrated. Figure 6 shows the localization of these two Tyr in the tertiary structure of the LCD and Supplementary Table S3 depicts some of the features of these Tyr such as rho, pKa, accessible surface area, and the surrounding amino acids that contribute to their pKa. The Tyr82 is a superficial residue located at a helix that participates in the interface between the subunits and it is at 13 Å distance from the carbonyl group of PLP. The low predicted pKa supports a high reactivity toward the formation of a tyrosyl radical that yields nitration (Wenk et al., 2021).

On the other hand, Tyr254 is absolutely preserved in Viridiplantae and it is located at an extended strand besides His237, at 7.3 Å from its Cα. Although experimental nitration data demonstrate a clear inhibition of LCD activity, the intimate mechanism of its inhibition remains still uncertain according to the available information.

### Modeling of pepper LCD provides additional insight into the active site and the catalytic mechanism

The best model of LCD from *C. annuum* L. was output by the Swiss Model server using as a template, not a cysteine desulfurase, but the structure of the PLP-dependent C-S lyase Egt2 from *Neurospora crassa* (PDB entry 5uts) (Irani et al., 2018). The structural superposition allowed the identification of Lys260 as the residue that forms the aldimine with the cofactor PLP and matches with residues involved in the mechanism of action of the PLP-dependent C-S lyase Egt2.

However, a closer inspection revealed important differences between the model of the LCD and 5uts, as expected from enzymes with different catalytic activity. In particular, the model LCD presents Phe147 at the position of Tyr134. Residue Tyr134 is strictly preserved in the closest homologs to 5uts, plays a dual role in both substrate binding and catalysis, and the mutation Y134/F yields a 5-fold increase in the *Km* and a reduction of *Kcat* to 40%. In addition, residues Arg75 and Phe343, which have been reported to sandwich the ergothioneine sulfenic acid intermediate, are not present in LCD.

The fact that the template used to model LCD from *C. annuum* L does not catalyze the desulfuration of L-Cys with the release of H_2_S and the presence of four cysteine residues surrounding the cofactor led us to evaluate the model from the perspective of a cysteine desulfurase. In general, cysteine desulfurases are grouped into type I and type II and a recognized structural difference between them is the catalytic loop that contains the nucleophilic Cys, being longer, more flexible, and not visible in the electron density in the type I enzymes. Hence, the comparison focused on the type II enzymes but neither the structural alignment nor the predicted pKa values support the role as a nucleophile of these Cys residues, discarding their participation in the catalysis.

As an alternative to investigate the residues comprising the active site, blind docking studies with the L-Cys and pyruvic acid, substrate, and product of the reaction catalyzed by LCD were conducted. In both cases, poses are located at two preferential zones, one of them being the active site. Their analysis reveals that His237 is at an interaction distance from the carboxylic group of both L-Cys and pyruvate, as well as the thiol group of L-Cys from the phosphate group of PLP. Interestingly, the keto group of the pyruvic acid is at 2 Å.

Thus, these docking results provide support for the identification of the active site and hypothesize the mechanism of catalysis. It is important to recall that from the point of view of chemistry, the transformation of L-Cys into pyruvate with the release of H_2_S and ammonia involves a β,γ-elimination (Vederas and Floss, 1980). In biological systems, the reaction is catalyzed by β-elimination enzymes, which are divided into two groups depending on whether the N1 of the bound PLP is protonated or not, and this feature determines the mechanism of the catalysis (Yamada et al., 2003).

**FIG. 8.**
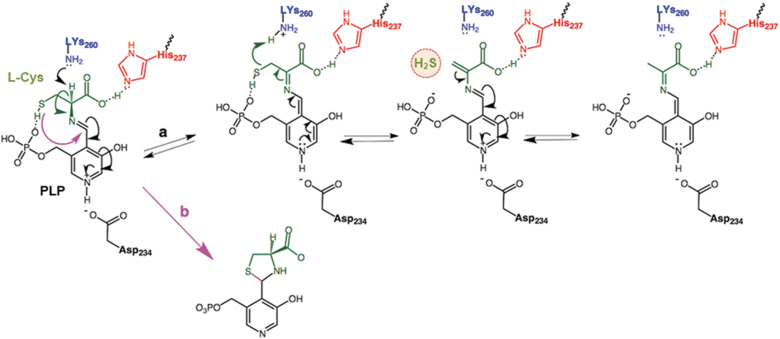
**Proposed mechanism of the transformation of L-Cys into pyruvate by the pepper LCD with the Lys260, His237, and PLP in the catalytic site of the enzyme with concomitant generation of H_2_S from L-Cys (path a), and the side reaction that leads to the inactivation of the enzyme (path b).** His237 is at an interaction distance from the carboxylic group of both L-Cys and pyruvate, as well as the thiol group of L-Cys from the phosphate group of PLP. On the other hand, the keto group of the pyruvic acid is at 2 Å. The transformation of L-Cys into pyruvate with the release of H_2_S and ammonia involves a β,γ-elimination where the N1 of the bound PLP is protonated and forms the quinone-like resonance structure, the α-hydrogen of the PLP-amino acid aldimine being readily abstracted by a basic residue. The model of LCD shows Asp234 at 2.6 Å, and the presence of its negatively charged amino acid allows to assume that N1 of the bound PLP is protonated. Once the aldimine (R–CH = N–R′) of the L-Cys is formed by displacement of Lys260, the α-hydrogen is abstracted. The hydrolysis of the aldimine of the amino acrylate yields pyruvic acid and ammonia and regenerates the internal aldimine between the carbonyl group of PLP and Lys260.

For type II β-elimination enzymes the N1 of the bound PLP is protonated and can form the quinone-like resonance structure, the α-hydrogen of the PLP-amino acid aldimine being readily abstracted by a basic residue. The model of LCD shows Asp234 at 2.6 Å, and the presence of this negatively charged amino acid leads us to assume that N1 of the bound PLP is protonated (Figs. 7A and 8, path a).

Once the aldimine of the L-Cys is formed by displacement of Lys260, the α-hydrogen is abstracted. According to the model, His259, Lys260, and His237 are the closest basic residues (Fig. 7A). The predicted pKa value His259 of around 3 supports that is not expected to be protonated but the distance to the α-hydrogen may be too far for a feasible attack. His237 is closer, but it is at a hydrogen bond distance from the carboxylic group of the L-Cys and probably its role is related to the positioning of the substrate rather than the abstraction of the proton (Fig. 7A).

However, on displacement by the substrate, Lys260 is released, and both its position and predicted pKa are consistent with its role in acid-base catalysis, abstracting the α-hydrogen and donating it to the sulfur atom to yield the H_2_S releasing group (Fig. 8, path a). The hydrolysis of the aldimine of the amino acrylate yields pyruvic acid and ammonia and regenerates the internal aldimine between the carbonyl group of PLP and Lys260.

The pre-treatment with 5 m*M* L-Cys or GSH yields the inhibition of the enzymatic activity. This result was not unexpected since the inhibition by high concentrations of L-Cys has been already reported for *Synechocystis* sp PCC 6803 desulfurases (Kato et al., 2000). In fact, it has been proposed that the inhibition is the consequence of a side reaction resulting from the attack of the nucleophilic sulfur to the Schiff base to yield a thiazolidine adduct, impeding the proton abstraction step necessary for the elimination reaction to proceed (Fig. 8, path b) (Koch, 2009). For the case of LCD, this hypothesis is plausible and explains the null effect of NaHS on the activity. At the physiological level, it might be a mechanism to avoid an overproduction of H_2_S that could be toxic for the cells.

## Conclusions

During the ripening of sweet pepper, two of the key enzymes involved in the generation of H_2_S, the cytosolic LCD and mitochondrial DCD, experimented a downregulation in their activity. However, the exogenous application of NO gas restores the activity, indicating that NO function upstream of H_2_S metabolism during ripening. On the other hand, *in vitro* analyses allow the evaluation of the effect of NO as well as ONOO^−^ on both types of H_2_S-generating enzymes.

Thus, whereas an *S*-nitrosation process seems to protect the LCD activity, the Tyr nitration mediated by ONOO^−^ exerts a drastic inhibition. Blind docking analyses provide additional insight into the implication of Lys260, His237, and PLP in the catalytic activity of the pepper LCD to generate H_2_S from L-Cys. Overall, NO exerts a dual effect on H_2_S metabolism during pepper fruit ripening depending on the level of NO and related species.

## Materials and Methods

### Pepper fruit samples and NO gas treatment

California sweet pepper (*C. annuum* L., cv. Melchor) fruits samples selection for the experimental plant materials and their subsequent NO treatments were established according to González-Gordo et al. (2019). Concisely, pepper fruits were selected from plants grown in plastic-covered experimental greenhouses (Syngenta Seeds, Ltd., Almería, Spain). The fruits lacking any external injuries at three developmental stages were categorized as green immature (G), BP1, and red ripe (R).

In the laboratory, the fruits were washed with distilled water and kept for 24 h at a low temperature (about 7°C ± 1°C). Then, the selected BP1 fruits were exposed to NO gas treatment (5 ppm NO for 1 h), as reported earlier. Supplementary Figure S1 shows a representative picture of the experimental design followed in this study with the representative phenotypes of sweet pepper fruits at different ripening stages and subjected to NO treatment (González-Gordo et al., 2019).

### Preparation of fruit crude extracts and protein concentration determination

Frozen samples of sweet pepper fruits at the different ripening stages and after NO treatment were powdered under liquid nitrogen using an IKA^®^ A11Basic analytical mill (IKA, Staufen, Germany) and then extracted in 100 m*M* Tris-HCl buffer, pH 7.5, containing 0.1% (v/v) Triton X-100, 1 m*M* ethylenediaminetetraacetic acid (EDTA), 1 m*M* MgCl₂, 5 m*M* ascorbate acid, and 10% (v/v) glycerol in a final 1:1 (w:v) plant material: buffer ratio. The obtained homogenates were centrifuged at 15,000 *g* for 30 min at 4°C, and the supernatants were used for enzymatic assays.

Protein concentration of the samples was determined with the Bio-Rad Protein Assay (Hercules, CA, USA) using bovine serum albumin as a standard.

### LCD activity spectrophotometric assay

LCD activity was measured spectrophotometrically by the release of H_2_S from L-Cys as previously described (Alvarez et al., 2010). Electronic laboratory notebook was not used.

### LCD and DCD activity on non-denaturing PAGE and image analyses

LCD and DCD isozymes were separated by non-denaturing PAGE on 8% and 6% acrylamide gels, respectively. Then, the gels were incubated in the following staining solution prepared in 100 m*M* Tris-HCl, pH 8.0 containing 20 m*M* L-Cys or D-Cys, 0.4 m*M* lead acetate, 50 μ*M* PLP hydrate, and 5 m*M* dithiothreitol. The generated H_2_S reacts with lead acetate to produce lead sulfide (PbS) forming dark brown precipitates corresponding to the enzymatic activity (Muñoz-Vargas et al., 2023). To corroborate the specificity of the isozymes, several internal controls were performed, one in the absence of the L-Cys or D-Cys substrate and another in the absence of the cofactor PLP/Vitamin B6 (Mooney and Hellmann, 2010). In both cases, the activity was not detected.

For image analyses of the band intensity of LCD and DCD isozymatic activities, the gels were photographed with a digital camera and the pictures were analyzed using the ImageJ 1.45 software throughout the plot line tool, which allows quantifying individually the area of the identified peaks associated with each band that corresponds to an isozymatic activity.

### *In vitro* treatment with NO donors, ONOO^−^, H_2_S, GSH, NO, and reducing agents

For the *in vitro* assays, samples from green pepper fruits were pre-incubated with different potential modulators, including SIN-1, a ONOO^−^ donor, and nitrating compound; GSNO and CysNO as NO donors; L-Cys and GSH, as reducing compounds; NaHS, as H_2_S donor; and KCN. In all cases, the solutions were freshly prepared before use at a concentration of 5 m*M*, and the treatments were done at 25°C for 1 h in the dark, except the treatment with SIN-1, which was at 37°C for 1 h.

### Expression and purification of cytosolic pepper LCD

The complementary DNA (cDNA) coding for the LCD (A0A2G3AJZ6; gene LOC107840630) with 1400 bp, which encodes a protein of 453 AA (molecular weight 51.45 kDa), was chemically synthesized to include a 6-His tag and cloned into a pET28 expression vector with optimization for expression in *E. coli*. Briefly, the recombinant protein was induced by adding IPTG to the medium, and bacteria were grown at 16°C for 16 h.

Then, the bacteria were harvested by centrifugation, lysed with native buffer, and centrifuged again. The supernatant corresponded to the native protein extract (NPE), whereas the pellet was solubilized with a denaturing buffer (urea 8 *M*) after centrifugation. The supernatant included the denatured protein extract (DPE). The purification of the recombinant LCD from the DPE was done by LYTRAP affinity chromatography *versus* His-Tag on Nickel resin equilibrated and binding with a phosphate buffered saline buffer, pH 7.5, then washed, and eluted by imidazole shift.

The obtained protein fractions were quantified by the Bradford method and analyzed by SDS-PAGE (12% acrylamide) with Coomassie blue staining. This procedure allowed us to obtain 0.64 μg LCD protein per μL.

### Identification of nitrated tyrosine in recombinant LCD by liquid chromatography and MS analysis (liquid chromatography electrospray ionization tandem mass spectrometric)

Purified LCD was individually digested with trypsin using a standard protocol. Briefly, 10 μg of precipitated recombinant protein was resuspended and denatured in 10 μL of 7 *M* urea, 2 *M* thiourea, 100 m*M* TEAB (triethylammonium bicarbonate), reduced with 5 m*M* Tris 2-carboxyethyl phosphine (TCEP; AB SCIEX, Foster City, CA), pH 8.0, at 37°C for 60 min, and followed by cysteine-blocking reagent chloroacetamide. Samples were diluted up to 50 μL with 25 m*M* TEAB to reduce the urea concentration. Sequence grade-modified trypsin (Pierce) was added to each sample (ratio 1:20 enzyme:sample), which was then incubated at 37°C overnight on a shaker. After digestion, samples were dried in a SpeedVac (Thermo Scientific, Waltham, MA).

Before MS analysis, sample cleaning was performed using ZipTip C18 tips (Millipore). Each sample was subjected to one-dimensional-nano LC ESI-MS/MS (Liquid Chromatography Electrospray Ionization Tandem Mass Spectrometric) analysis using an Ultimate 3000 nano HPLC system (Thermo Fisher Scientific) coupled online to an Orbitrap Exploris 240 mass spectrometer (Thermo Fisher Scientific).

Peptides were eluted onto a 15 cm × 75 μm Easy-spray PepMap C18 analytical column at 45°C and separated at a flow rate of 300 nL/min using a 40 min gradient ranging from 2% to 35% mobile phase B (mobile phase A: 0.1% formic acid [FA]; mobile phase B: 80% acetonitrile [ACN] in 0.1% FA). The loading solvent was 2% ACN in 0.1% FA, and the injection volume was 5 μL.

Data acquisition was performed using a data-dependent top-20 method, in full scan positive mode, scanning 375–1200 m/z. Survey scans were acquired at a resolution of 60,000 at m/z 200, with Normalized Automatic Gain Control (AGC) target (%) of 300 and a maximum injection time (IT) in AUTO. The top 20 most intense ions from each MS1 scan were selected and fragmented *via* higher-energy collisional dissociation (HCD).

Resolution for HCD spectra was set to 45,000 at m/z 200, with AGC target of 100 and a maximum ion IT in AUTO. Isolation of precursors was performed with a window of 0.7 m/z, exclusion duration (s) of 5 s and the HCD collision energy was 30. Precursor ions with single, unassigned, or six and higher charge states from fragmentation selection were excluded (Hung et al., 2007).

### Proteomics data analysis and sequence search

Raw instrument files were converted to mgf files, and MS/MS spectra were searched using Mascot v2.8 against a composite target database built from the *C. annuum* reference proteome downloaded from UniprotKB containing 35548 sequences plus a collection of common laboratory contaminants. The search engine was configured to match potential peptide candidates with a mass error tolerance of 10 ppm and fragment ion tolerance of 0.025 Da, allowing for up to two missed tryptic cleavage sites, considering fixed carbamidomethyl modification of cysteine and variable oxidation of methionine, deamidated N or Q, acetylation at Protein N-term, and Nitro modification of tyrosine. The Mascot cut-off value was considered to be statistically significant peptides. Manual validation of MS2 spectra was performed for every nitro-modified peptide candidate.

### Protein modeling and *in silico* analyses

The tertiary structure of the LCD from *C. annuum* L. (UniProtKB entry A0A1U8DYD9) (UniProt Consortium, 2008) was modeled at M4T server (Fernandez-Fuentes et al., 2007), I-Tasser server 3D (Zhang, 2008) Swiss Model Server (Arnold et al., 2006), Phyre2 server (Kelley and Sternberg, 2009), and RaptorX server (Källberg et al., 2012). The alignment of protein sequences was done with the MUSCLE computer program (Edgar, 2004). Model quality was evaluated by the distribution of atom–atom contacts (Errat method) (Colovos and Yeates, 1993), three-dimensional profiles (Verify3D) (Eisenberg et al., 1997), Procheck (Laskowski et al., 1993), and Qmean4 and Qmean-Z score (Benkert et al., 2011; Benkert et al., 2008). WoLF PSORT program was used for protein subcellular location prediction (Horton et al., 2007).

Coordinates were inspected with Chimera (Pettersen et al., 2004), the solvent accessibility was estimated with DSSP (Kabsch and Sander, 1983), and the pKa values were predicted with PROPAKA 3.4 (Li et al., 2005).

### Blind docking

The coordinates of the structure of LCD from *C. annuum* L. computed at the Swiss Model Server were prepared for docking with Dock Prep, a tool implemented in Chimera (Pettersen et al., 2004). Docking with the coordinates of L-Cys and pyruvate taken from the ZINC database (Sterling and Irwin, 2015) was carried out at Swiss-Dock sever (Grosdidier et al., 2011) in an accurate mode, allowing flexibility for the side chains within 5 Å of any atom of the ligand in its reference binding mode, without defining the region of interest (blind docking).

Results were sorted by their full fitness score, a parameter that accounts for the total energy of the system, including the solvation-free energy (Zoete et al., 2010), and analyzed with Chimera (Pettersen et al., 2004).

Molecular evolution studies were carried out at the Evolutionary Trace server (Mihalek et al., 2004) using as input the structure of LCD from *C. annuum* L. computed at the Swiss Model and a BLASTP (Altschul et al., 1997) search on the UniProtKB (UniProt Consortium, 2008) Viridiplantae consisting of 500 sequences. The evolutionary conservation was ranked according to the rho parameter that deviates from 1 as the variability (*i.e*., less evolutionary importance) increases (Mihalek et al., 2004).

## Supplementary Material

Supplemental data

Supplemental data

Supplemental data

Supplemental data

Supplemental data

Supplemental data

Supplemental data

Supplemental data

Supplemental data

Supplemental data

## Supplementary Material

Supplementary Table S3

ImmunoblotUncroppedRawImage

## Abbreviations Used

% ID: percentage of identity in comparison to the corresponding Arabidopsis protein
AA: amino acid
ACN: acetonitrile
AGC: Automatic Gain Control
BP: breaking point
Cys: cysteine
CysNO: *S*-nitrosocysteine
Cyt: cytosol
DCD: D-cysteine desulfhydrase
DPE: denatured protein extract
FA: formic acid
FT: flow through
GSH: reduced glutathione
GSNO: *S*-nitrosoglutathione
H_2_S: hydrogen sulfide
HCD: higher-energy collisional dissociation
IN: input
IT: injection time
KCN: potassium cyanide
L-Cys: L-cysteine
LCD: L-cysteine desulfhydrase
Mit: mitochondrion
MS: mass spectrometry
MW: molecular weight
NaHS: sodium hydrosulfide
NO: nitric oxide
NO_2_: nitrogen dioxide
ONOO^−^: peroxynitrite
PAGE: polyacrylamide gel electrophoresis
Pla: plastid
PLP: pyridoxal 5′-phosphate
PTM: post-translational modification
RMSD: root mean square deviation
RNS: reactive nitrogen species
SDS: sodium dodecyl sulphate
SEM: standard error of the mean
SIN-1: 3-morpholinosydnonimine
TEAB: triethylammonium bicarbonate
Tyr: tyrosine
