# Supplementary material for: H2S-Generating Cytosolic L-Cysteine Desulfhydrase and Mitochondrial D-Cysteine Desulfhydrase from Sweet Pepper (Capsicum annuum L.) Are Regulated During Fruit Ripening and by Nitric Oxide
Source: Antioxid Redox Signal. 2023 Jul 17;39(1-3):2–18. doi: 10.1089/ars.2022.0222 (PMC10585658; doi:10.1089/ars.2022.0222)
Supplement: Supplemental data [file Supp_FigS1.docx]

**
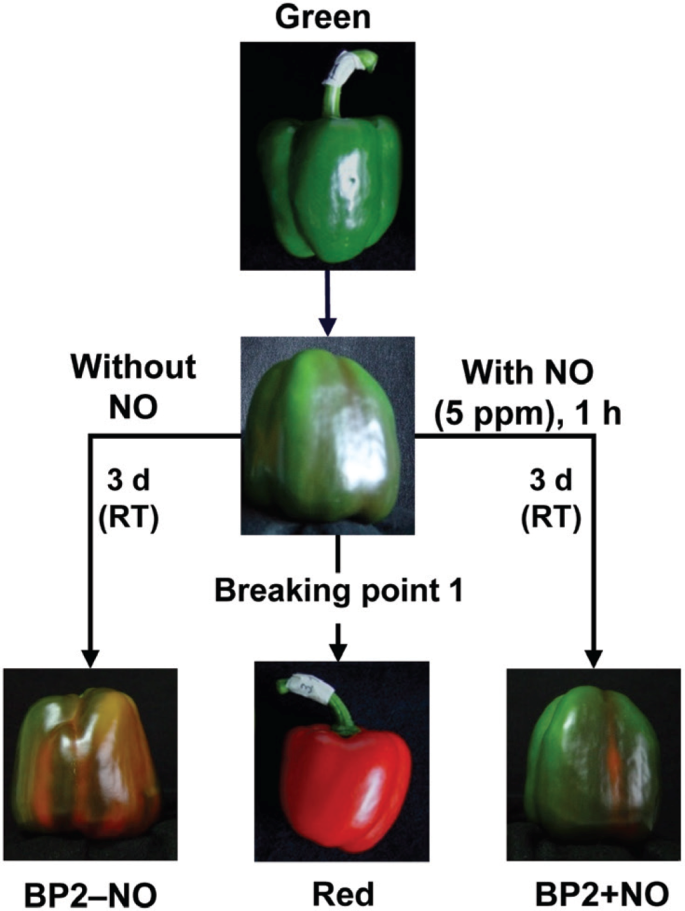
**

**Figure S1.** Representative picture of the experimental design used in this study with the phenotypes of sweet pepper (*Capsicum annuum* L.) fruits at different ripening stages and treatments: immature green, breaking point 1 (BP1), breaking point 2 without nitric oxide (NO) treatment (BP2 – NO), breaking point 2 with NO treatment (BP2 + NO), and ripe red. Pepper fruits were subjected to a NO-enriched atmosphere (5 ppm) in a methacrylate box for one hour and were then stored at room temperature (RT) for 3 days. Reproduced with permission from González-Gordo et al. (2022).
