# Supplementary material for: H2S-Generating Cytosolic L-Cysteine Desulfhydrase and Mitochondrial D-Cysteine Desulfhydrase from Sweet Pepper (Capsicum annuum L.) Are Regulated During Fruit Ripening and by Nitric Oxide
Source: Antioxid Redox Signal. 2023 Jul 17;39(1-3):2–18. doi: 10.1089/ars.2022.0222 (PMC10585658; doi:10.1089/ars.2022.0222)
Supplement: Supplemental data [file Supp_TableS1.docx]

**Table S1.** Evaluation of the quality of the models of the structure of pepper LCD computed by different servers.

| **Sever** | | **Qmean-Z ^a^** | **Qmean4^b^** | **Errat** | **Verify3D** | **Procheck^a^** | **Coverage** | **Templates (identity)** |
| --- | --- | --- | --- | --- | --- | --- | --- | --- |
| **M4T** | | -3.708 | 0.643 | 55.2632 | 88.3% | 88.1% (0,0%) | 45-430  (85%) | **5v1x** (27.92%) |
| **ITasser** | #1 | -9.546 | 0.458 | 78.427 | 81.90% | 69.5% (1.5%) | 1-453 (100%) | 5utsA (27%); 7dlwA (17%); 1jf9 (22%); 6wciA (22%); 5j8qA (20%); 6c9eA (21%); 7nhaB (22%); 6a6eA (18%) |
|  | #2 | -11.560 | 0.392 | 77.9775 | 88.52% ) | 67.5% (3.0%) |  |  |
|  | #3 | -8.057 | 0.506 | 87.8652 | 88.96% () | 72.2% (2.5%) |  |  |
|  | #4 | -9.555 | 0.457 | 83.2957 | 81.68% | 68.2% (1.8%) |  |  |
|  | #5 | -10.880 | 0.414 | 82.4719 | 81.24% | 67.0% (2.2%) |  |  |
| **Swiss** | | -2.894 | 0.662 | 84.2893 | 94.6%) | 91.1% (0.1%) | 33-447 (87%) | 5uts (27%) |
| **Phyre2** | #1 | -5.704 | 0.571 | 51.3648 | 75.18 **l**) | 85.2% (1.1%) | 29-443 (91%) | 5utsA (27%) |
|  | #2 | -6.233 | 0.531 | 53.7084 | 83.79% | 87.7% (1.4%) | 34-443 (90%) | d1j9A (20%) |
|  | #3 | -8.255 | 0.482 | 48.9796 | 79.75 | 83.5% (1.7%) | 34-443 (90%) | C5b87B (20%) |
|  | #4 | -6.801 | 0.533 | 56.1881 | 75.73 | 84.8% (1.4%) | 32-443 (90%) | D1t3ia (19%) |
| **RaptorX** | #1 | -4.071 | 0.636 | 79.8627 | 78.38 | 80.5% (1.8%) | 1-453  (100%) | Distance-based Protein Folding Powered by Deep Learning^d^ |
|  | #2 | -6.836 | 0.531 | 88.0668 | 88.30% | 82.5% (0.8%) |  |  |
|  | #3 | -4.283 | 0.629 | 84.7575 | 93.16% | 78.2% (1.2%) |  |  |
|  | #4 | -3.090 | 0.668 | 69.9774 | 81.24% | 82.8% (0.5%) |  |  |
|  | **#5** | -2.089 | 0.701 | 80.2752 | 88.08% | 85% (1.5%) |  |  |

**^a^** Absolute quality that directly indicates how many standard deviations the model's QMEAN score differs from expected values for experimental structures (i.e."degree of nativeness")

**^b^** Model reliability ranging from 0 to 1

^c^ Percentage of residues in most favored regions of the Ramachandran Plot and in brackets the percentage of residues in disallowed regions

^d^ Jinbo Xu, Matthew McPartlon, and Jin Li, “Improved Protein Structure Prediction by Deep Learning Irrespective of Co-Evolution Information,” *Nature Machine Intelligence* 3, no. 7 (July 2021): 601–9, doi:10.1038/s42256-021-00348-5.
