# Supplementary material for: H2S-Generating Cytosolic L-Cysteine Desulfhydrase and Mitochondrial D-Cysteine Desulfhydrase from Sweet Pepper (Capsicum annuum L.) Are Regulated During Fruit Ripening and by Nitric Oxide
Source: Antioxid Redox Signal. 2023 Jul 17;39(1-3):2–18. doi: 10.1089/ars.2022.0222 (PMC10585658; doi:10.1089/ars.2022.0222)
Supplement: Supplemental data [file Supp_FigS2.docx]

**
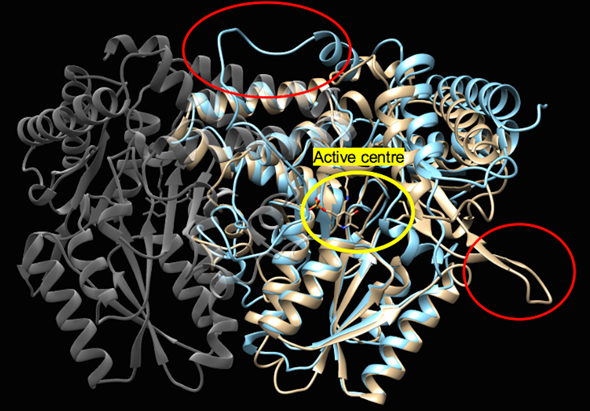
**

**Fig S2.** Structural superposition of the models of LCD from *Capsicum annuum* computed at Swiss Model server (cyan and gray) and RaptorX server (dark goldenrod). Regions with major differences between both models are encircled in red and the active center in yellow.
