# Supplementary material for: H2S-Generating Cytosolic L-Cysteine Desulfhydrase and Mitochondrial D-Cysteine Desulfhydrase from Sweet Pepper (Capsicum annuum L.) Are Regulated During Fruit Ripening and by Nitric Oxide
Source: Antioxid Redox Signal. 2023 Jul 17;39(1-3):2–18. doi: 10.1089/ars.2022.0222 (PMC10585658; doi:10.1089/ars.2022.0222)
Supplement: Supplemental data [file Supp_FigS3.docx]

**
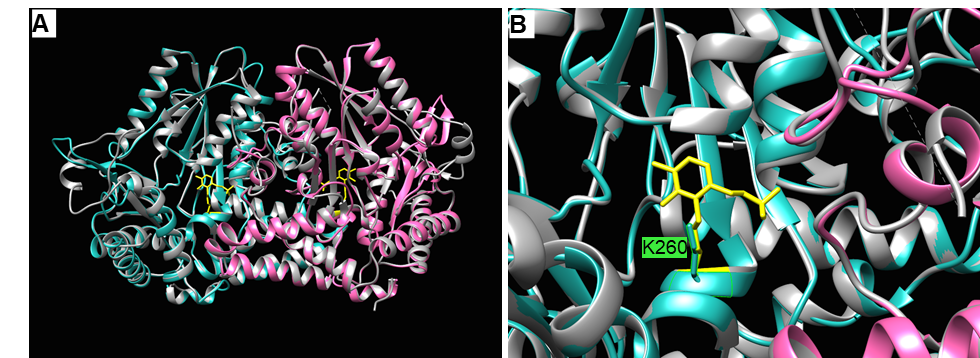
**

**Fig S3. A.** Structural superposition of the template 5uts (gray) and the model of LCD computed at Swiss Model server (subunits are shown in light green and hot pink). **B.** Detail of the region of 5uts comprising PLP (yellow) and identification of Lys260 as the residue of the model that forms the aldimine.
