# Supplementary material for: H2S-Generating Cytosolic L-Cysteine Desulfhydrase and Mitochondrial D-Cysteine Desulfhydrase from Sweet Pepper (Capsicum annuum L.) Are Regulated During Fruit Ripening and by Nitric Oxide
Source: Antioxid Redox Signal. 2023 Jul 17;39(1-3):2–18. doi: 10.1089/ars.2022.0222 (PMC10585658; doi:10.1089/ars.2022.0222)
Supplement: Supplemental data [file Supp_FigS4.docx]

**
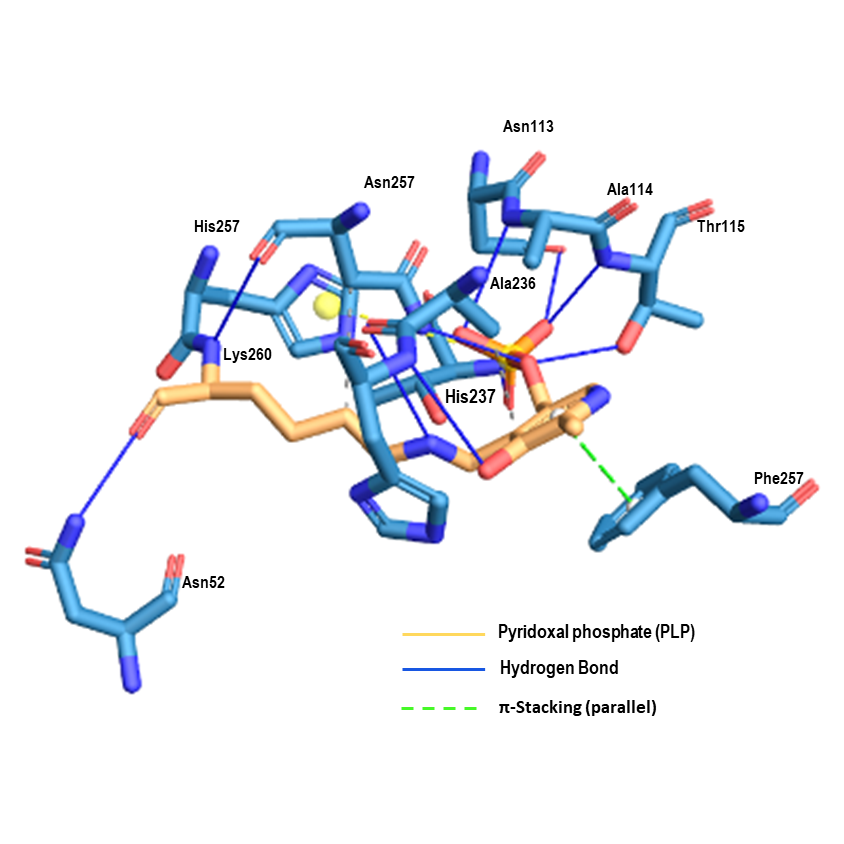
**

**Figure S4.** Interactions of the cofactor pyridoxal-5’-phosphate (PLP) forming an aldimine with Lys260 (in sapphire color) with the surrounding residues (in cobalt color) as depicted by PLIP server. Lys260 establishes hydrophobic interactions with Ala236, π-stacking with Phe257, hydrogen bonds with Asn52, Asn113, Ala114, Thr115, His237, and Thr304, and salt bridges with His259.
