# Supplementary material for: H2S-Generating Cytosolic L-Cysteine Desulfhydrase and Mitochondrial D-Cysteine Desulfhydrase from Sweet Pepper (Capsicum annuum L.) Are Regulated During Fruit Ripening and by Nitric Oxide
Source: Antioxid Redox Signal. 2023 Jul 17;39(1-3):2–18. doi: 10.1089/ars.2022.0222 (PMC10585658; doi:10.1089/ars.2022.0222)
Supplement: Supplemental data [file Supp_FigS5.docx]

**Figure S5. A.** Cys residues close to K260 according to the LCD model. Subunit B is shown in dark gray. **B.** Detail of the Cys residues closed to the cofactor (green) as modeled by the Swiss Model (red) and RaptorX (blue). **C.** Structural superposition of the model of LCD (in red and Cys in yellow) with type II cysteine desulfurase from *E. coli* (PDB entries 1c0n and qkmj in gray and tea green respectively), from *B. subtilis* (PDB entry 5zs9 in magenta) and *A. thaliana* (cyan) showing the catalytic Cys shadowed in magenta. **D.** Detail of the catalytic loop of the enzyme from *A. thaliana* (encircled in yellow), showing C384 and the equivalent region in LCD model (encircled in gray).
