# Supplementary material for: H2S-Generating Cytosolic L-Cysteine Desulfhydrase and Mitochondrial D-Cysteine Desulfhydrase from Sweet Pepper (Capsicum annuum L.) Are Regulated During Fruit Ripening and by Nitric Oxide
Source: Antioxid Redox Signal. 2023 Jul 17;39(1-3):2–18. doi: 10.1089/ars.2022.0222 (PMC10585658; doi:10.1089/ars.2022.0222)
Supplement: Supplemental data [file Supp_TableS2.docx]

**Table S2.** Main features of the Lys260 and the potential catalytic cysteines.

| **Residue** | **Rho^1^** | **pKa** | **Residues contributing to pKa^2^** | **ASA (Å^2^)** |
| --- | --- | --- | --- | --- |
| Lys260 | 1.62 | 7.69  7.43 | D204, C211, D234, R418  D204, C211, D234, R418 | 12  12 |
| Cys58 | 3.51 | 12.72  12.70 | R49, D306  R49, D306 | 1  0 |
| Cys145 | 5.08 | 11.54  12.19 | K151, D204, D358, E401  D204, C355, E401 | 26  19 |
| Cys211 | 20.07 | 12.00  12.03 | D204, K260, R418  K260, R418 | 0  0 |
| Cys264 | 6.86 | 14.00  14.12 | C58  C58 | 1  1 |

^1^ Deviates from 1 as the variability (i.e. less evolutionary importance) increases

^2^ Residues interacting with the target residue and affecting its pka
