# Supplementary material for: H2S-Generating Cytosolic L-Cysteine Desulfhydrase and Mitochondrial D-Cysteine Desulfhydrase from Sweet Pepper (Capsicum annuum L.) Are Regulated During Fruit Ripening and by Nitric Oxide
Source: Antioxid Redox Signal. 2023 Jul 17;39(1-3):2–18. doi: 10.1089/ars.2022.0222 (PMC10585658; doi:10.1089/ars.2022.0222)
Supplement: Supplemental data [file Supp_FigS6.docx]

**Figure S6.** Blind docking of Cys (**A and B**) and pyruvate (**C and D**) on the model of pepper fruit LCD. In A and C, only the results of the subunit with the surface colored according to the Kyte-Doolittle scale (from dodger blue for the most hydrophilic to white at 0.0 to orange-red for the most hydrophobic) are shown. B and D detail the poses at the active center.
