# Supplementary material for: H2S-Generating Cytosolic L-Cysteine Desulfhydrase and Mitochondrial D-Cysteine Desulfhydrase from Sweet Pepper (Capsicum annuum L.) Are Regulated During Fruit Ripening and by Nitric Oxide
Source: Antioxid Redox Signal. 2023 Jul 17;39(1-3):2–18. doi: 10.1089/ars.2022.0222 (PMC10585658; doi:10.1089/ars.2022.0222)
Supplement: Supplemental data [file Supp_TableS3.docx]

**Table S3.** Main features of the nitrated tyrosine residues. ASA, accessible surface area.

| **Residue** | **Rho^1^** | **pKa** | **Residues contributing to pKa^2^** | **ASA (Å^2^)** |
| --- | --- | --- | --- | --- |
| Tyr82 | 20.39 | 9.84  9.39 | E290, R384, R388, E394  E290, R384, R388, E394 | 110 |
| Tyr254 | 1.00 | 12.99  13.11 | D234, D245, S256, R331  D234, D245, S256, R331 | 7  6 |

^1^ Deviates from 1 as the variability (i.e. less evolutionary importance) increases

^2^ Residues interacting with the target residue and affecting its pKa
